# Multi-omics analysis reveals focal adhesion characteristic associated tumor immune microenvironment in colon adenocarcinoma

**DOI:** 10.3389/fgene.2023.1088091

**Published:** 2023-03-06

**Authors:** Xiaoming Xu, Jingzhi Wang

**Affiliations:** ^1^ Department of Gastroenterology, Jining First People’s Hospital, Jining, China; ^2^ Department of Radiotherapy Oncology, The Affiliated Yancheng First Hospital of Nanjing University Medical School, The First People’s Hospital of Yancheng, Yancheng, China

**Keywords:** focal adhesion genes, gene signature, colon cancer, GEO database, TCGA data base

## Abstract

Colon adenocarcinoma (COAD) is one of the most frequent malignant lesions of the digestive system in humans, with an insidious onset. At the time of diagnosis, most of them have developed to the middle and late stages, and cancer cells have metastasized, and the prognosis is poor. Treatment options for progressive COAD are limited, and despite the promise of immunotherapy, immunotherapy response rates are low. The assembly and disaggregation of focal adhesion are critical for the directional migration of tumor cells to different sites, and it is unclear whether focal adhesion-related genes are involved in the development and prognosis of colon adenocarcinoma. This study aimed to investigate the role of focal adhesion genes in the occurrence and prognosis of COAD. We obtained datasets of COAD patients, including RNA-sequencing data and clinical information, from the TCGA and GEO databases (GSE17538 and GSE39582). Through CNMF clustering, two molecular subtypes with different expression patterns of focal adhesion genes were identified, and it was found that the molecular subtype with low expression of focal adhesion genes had better prognosis. Then the prediction signature was constructed by LASSO-Cox regression model, and the receiver operating characteristic (ROC) curve showed that the 4-gene signature had a good prediction effect on COAD 1-, 2-, and 3-year OS. Gene function enrichment analysis showed that the high-risk group was mainly enriched in immune and adhesion-related signaling pathways, suggesting that focal adhesion genes may affect the development and prognosis of COAD by regulating the immune microenvironment and tumor metastasis. The interaction between focal adhesion genes and immunity during the occurrence of COAD may help improve the response rate of immunotherapy, which also provides new ideas for the molecular mechanism and targeted therapy in COAD.

## 1 Introduction

Colorectal cancer is one of the most frequent malignant lesions of digestive system in humans, with the highest incidence in the age group of 40–50 years old. About 41% occur in the proximal colon, about 22% involve the distal colon, and about 28% invade the rectum (Keum and Giovannucci, 2019). Colorectal cancer is one of the diseases with the highest incidence and mortality of cancer in the world. The new cases of colorectal cancer in the United States in 2002 were 104,610, accounting for 8%–9% of all cancers, and the number of deaths was 53,200 (Siegel et al., 2020). A rising global burden of colorectal cancer which persists until the year 2040 and likely beyond (Morgan et al., 2023). Among colorectal cancer, COAD is the most common. Although significant progress has been made in the treatment of COAD in recent years, including surgical resection, radiotherapy, chemotherapy and molecular targeted therapy, the incidence and mortality of COAD are still increasing year by year, and the prognosis of patients with advanced COAD is far from satisfactory (Chuang et al., 2022b). Therefore, it is of great importance to further study the molecular mechanism behind the occurrence and development of COAD in order to reveal new prognostic biomarkers and therapeutic targets.

The adhesion between cells and the extracellular matrix is achieved through focal adhesions. In COAD patients, integrins and focal adhesion hub signaling networks have vastly overlapping mechanisms. The activation of FAK is likely contributed to by β4 integrin inducing a conformational change in the FAK autoinhibitory intramolecular interaction through interaction with the linker region that contains the 25aa motif, this will lead to contributes to tumorigenicity of COAD (Tai et al., 2016). The alpha1-integrins enhances the aggressiveness of colon cancer cells by activating the FAK-src molecular scaffolds (Van Slambrouck et al., 2007). Focal adhesions are micron-sized integrin-mediated adhesion complexes (focal complex), which can fix cells on the extracellular matrix and connect the extracellular matrix, adhesion molecules and cytoskeleton; on the other hand, focal adhesions can sense external mechanical stimuli and transmit signals into cells (Mierke, 2019). And participate in adhesion-mediated signaling pathways, cell migration and invasion, extracellular matrix remodeling, tissue formation and other physiological activities. A variety of focal adhesion proteins have been identified (Schumacher et al., 2022). Some of them bind integrin (Li et al., 2021), some bind to cytoskeletal proteins (Wang et al., 2022), and some bind to cell membrane lipid molecules (Senju and Lappalainen, 2019). Among these proteins, some are known to be involved in tumor initiation and progression. For example, focal adhesion kinase (FAK) has been reported to be critical in tumors (Zhang et al., 2022). FAK is a non-receptor tyrosine kinase that is overexpressed and activated in tumor cells (Pomella et al., 2022). FAK can transmit and sense mechanical signals, and regulate the tumor immune microenvironment. The expression and activation of FAK are often related to the survival, proliferation and migration of endothelial cells, and play an important role in tumorigenesis (Chuang et al., 2022a). FAK is involved in the regulation of TAF, and tumor-derived lysyl oxidase-like protein 2 can activate fibroblasts through integrin-mediated FAK activation and AKT signaling (Barker et al., 2013). Focal adhesions can regulate immune cell behavior in tumors. M1-type macrophages can promote the metastasis of hepatocellular carcinoma by activating the expression of FAK/NF-κB (Chen et al., 2014). Integrin-mediated macrophage motility requires FAK signaling, and FAK inhibitors reduce macrophage infiltration into tumor tissue. These results suggest that focal adhesions mediate complex signaling network pathways, which can directly regulate tumor cell adhesion, proliferation, growth, survival, angiogenesis, invasion, and metastasis, as well as indirectly promote tumorigenesis and metastasis by regulating the tumor microenvironment. However, the specific regulatory mechanism of focal adhesions in colon adenocarcinoma remains to be further studied.

In this study, in order to systematically study the function of focal adhesions in colon adenocarcinoma, 22 prognosis-related focal adhesion genes were collected, the expression changes and functional mechanisms of focal adhesion genes were analyzed in colon adenocarcinoma transcriptome samples, combined with Prognostic clinical information of colon adenocarcinoma patients, we adopted a novel rank-based pairwise comparison algorithm to select effective focal adhesion pairs and established a signature. After systematic analysis, 4-gene signature has high accuracy in determining the survival time of patients, and is an independent prognostic factor for COAD. In addition, its remodeling effect on the tumor microenvironment provides new clues for the molecular mechanism and targeted therapy of focal adhesions.

## 2 Materials and methods

### 2.1 Data and preprocessing

First, we downloaded COAD patients’ HTSeq-FPKM gene expression data and related clinical information from the TCGA database as a training set. After excluding patients with incomplete survival data, a total of 343 COAD patients with complete follow-up data and a follow-up time of more than 30 days were included. In the further validation process, we adopted the same inclusion criteria, and downloaded the dataset GSE17538 from the GEO database to include 204 COAD patients, and downloaded the dataset GSE39582 to include a total of 523 COAD patients. The possibility of batch effects due to abiotic biases between the TCGA-COAD dataset and the GSE17538, GSE39582 datasets was reduced by the “Combat” algorithm of the R package “sva” (Johnson et al., 2007). 199 focal adhesion genes were retrieved from the MSigDB database (https://www.gsea-msigdb.org/gsea/msigdb). Immunohistochemical data for CAV2, FLT1, THBS3 and VAV3 in tumor and control tissues were obtained from https://www.proteinatlas.org/.

### 2.2 Identification of molecular subtypes

The COAD samples were clustered using the CNMF clustering algorithm. 199 focal adhesion genes were selected for subsequent screening. First, Cox survival regression analysis was performed on focal adhesion-related genes significantly associated with OS time using the survival R package (*p* < 0.001), followed by CNMF clustering in the COAD cohort using prognosis-associated genes were identified through “CancerSubtypes” R Software packages (Xu et al., 2017). Combined profile coefficient and elbow coefficient to determine the number of new subtypes (K values). Kaplan-Meier survival analysis and log-rank test were performed on adhesion molecules subtypes using the “survival” and “survminer” packages. At the same time, dimensionality reduction analysis was performed on the mRNA expression data of the above candidate genes, and the subtype distribution was verified by principal component analysis (PCA). We also quantified the PCA scores for each patient. PCA Score = PC1-PC2. We compared PCA scoring differences in subgroups as well as patient survival status to reflect subgroup heterogeneity.

### 2.3 Construction, validation and application of focal adhesion gene pair signatures

Taking the COAD patients of TCGA as the training set, ARGs were incorporated into Lasso-Cox regression analysis for further screening and removal of collinearity using the “glmnet” package, the penalized COX regression was implemented using the glmnet function and 10-fold cross-validation was performed using the cv. glmnet function (Friedman et al., 2010; Simon et al., 2011). The minimum likelihood deviation (the minimum likelihood deviation corresponding to the best λ value obtained) estimated from the 10-fold cross-validation in the metadata set is used to determine the penalty parameters, and the coefficient *ß* (Coef) values of each feature are extracted under the specified lambda. A prognostic risk score model with *ß* (Coef) values multiplied by ARLP scoring was finally established. Risk Score = Coef1 × ARLP1 + Coef2 × ARLP2 + Coef3 × ARLP3 +…+ Coefn × ARLPn. We divided 343 patients into high-risk and low-risk groups using the median risk score as the threshold value. Kaplan–Meier (K-M) analysis, log rank test and ROC were performed to further assess the stability of the model. Univariate and multifactor cox regressions were performed for clinical traits and signature to clarify the independence of signature from other clinical traits.

### 2.4 Functional enrichment analysis

Gene Set Variation Analysis (GSVA) is a kind of Gene Set enrichment method, which calculates the estimated proportion of certain pathways or signatures in different clusters according to the expression spectrum, obtains related metabolic and carcinogenic pathway Gene sets for enrichment Analysis through previous studies. In addition, H. all. v7.4. symbols were downloaded from the molecular characteristics database (MSigDB), and the same GSVA method was used for enrichment analysis.

Based on the GO (BP, MF, CC) Reactome Hallmark gene set, we conducted a gene set enrichment study (GSEA) on the high-risk and low-risk groups in order to explain the influence of mutations on the occurrence and development of COAD. APC (genes with the highest mutation frequency) differed significantly in high and low risk groups. To explore the effect of mutations on the development of COAD, we likewise performed GSEA analysis on APC mutant and wild type to clarify which mechanisms are activated or suppressed in the mutant phenotype compared to the wild type.

### 2.5 Tumor microenvironment analysis

In immune cell analysis for immune cell infiltration and tumor mutation load estimation, different algorithms were used simultaneously, such as MCPcounter (Becht et al., 2016), CIBERSORT (Newman et al., 2015), XCell (Aran et al., 2017), TIMER (Li et al., 2017), EPIC (van Veldhoven et al., 2011), Cibersort-ABS (Tamminga et al., 2020), QUANTISEQ (Finotello et al., 2019). We compared the differences in immune infiltration between high and low risk groups. We also calculated the correlation between gene expression in the model and immune cells, which we included only when the two software considered a correlation. In addition, the ssGSEA algorithm was used to estimate immune cells and immune-related functions. The ESTIMATE algorithm was used to calculate the immune score to reflect the state of the microenvironment.

### 2.6 Signature application

Due to immune therapy is a newly used treatment method for COAD, we use the microsatellite instability (MSI), human leukocyte antigen (HLA) diversity, immune checkpoint expression to determine the choice of immunotherapy in high and low risk group of patients.

## 3 Results

### 3.1 CNMF determined two subtypes of COAD

**FIGURE 1:**
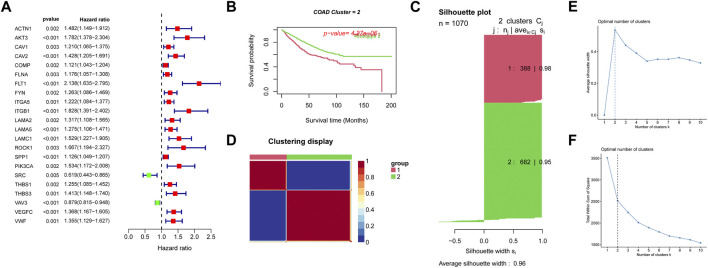
Subgroups of COAD defined by the most significant adhesion molecules related genes filter by univariate cox analysis. **(A)** Forest plot showing the 22 the most significant adhesion molecules screened by univariate cox analysis. **(B)** OS curves based on COAD patients for the two adhesion molecules related clusters. **(C)** Contour coefficients and mean contour coefficients of the two clusters, contour values are a measure of how similar an object is to its own cluster (cohesion) compared to other clusters (separation). **(D)** Heatmap of the sample similarity matrix visualizing the difference between the two clusters. **(E)** The change of contour coefficients as the clusters increases. **(F)** Variation of elbow coefficients as the clusters increase.

We retrieved 199 focal adhesion genes in our MSigDB database (https://www.gsea-msigdb.org/gsea/msigdb). Univariate cox analysis for focal adhesion genes in the TCGA-COAD dataset and GSE17538, GSE39582 datasets with *p*-value <0.005 was performed, and 22 focal adhesion genes associated with prognosis were identified (Figure 1A). To further clarify the prognostic value of focal adhesion genes, we performed CNMF clustering to identify new subtypes. Kaplan-Meier survival analysis and log-rank test suggested that the COAD samples could be divided into two groups (Figure 1B). Consistent NMF was again performed to define 2 subtypes, C1 (*n* = 388), C2 (*n* = 682), with an average silhouette width of 0.96 (Figure 1C). The adjusted sequential sample similarity matrix heat map Figure 1D shows that the optimal value of k was determined when the contour coefficient was maximum (Figure 1E) and the decrease in elbow coefficient (Figure 1F) started to level off, k = 2 was the optimal number of clusters.

### 3.2 Different expression patterns of adhesion spot-associated genes in the two isoforms

**FIGURE 2:**
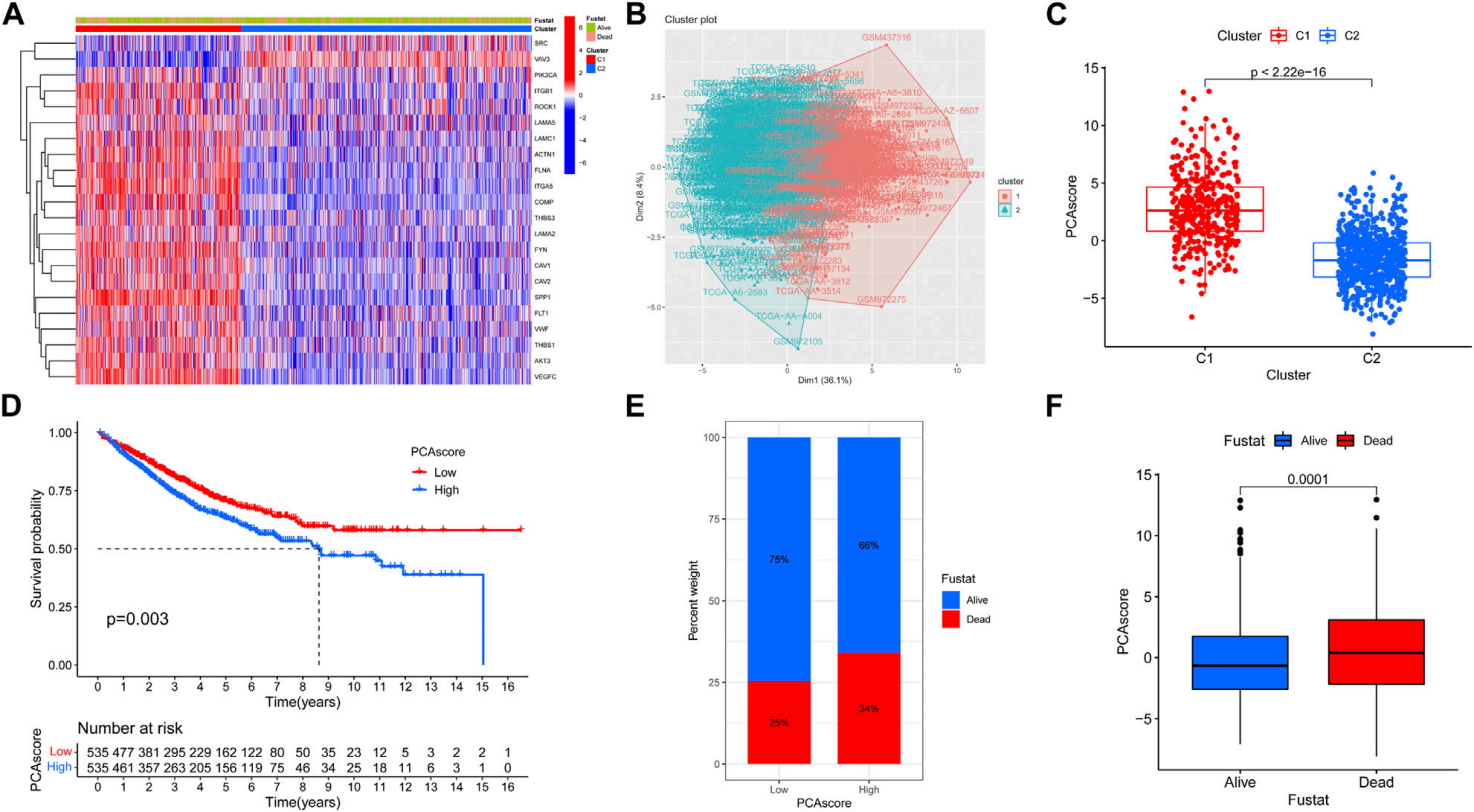
Evaluate the degree of differentiation of clustering. **(A)** Heat map showing the expression differences of 22 genes in the two isoforms. **(B)** Fviz cluster function showing the Kmeans clustering results of the 2 isoforms as a scatter plot in 2D space. **(C)** Wilcox difference analysis of PCA score for C1 and C2 subtypes. **(D)** OS curves based on COAD patients for the high PCA score group and low PCA score group. **(E)** Differences in survival status between high PCA score group and low PCA score group. **(F)** Differences in PCA score between the Alive group and the Dead group.

We also compared the expression of focal adhesion genes between different subtypes, except for the SRC and VAV3 genes, the other 20 focal adhesion genes were more highly expressed in C1 compared to C2 subtypes (Figure 2A). PCA analysis was further dimensionally reduced to visualize differences in expression patterns of focal adhesion genes (Figure 2B). We also quantified the PCA score of each patient, PCA Score = PC1-PC2. Among them, the PCA score of the C1 subgroup was significantly higher than that of the C2 subgroup. Survival analysis showed that patients with high PCA scores had significantly lower OS compared with patients with low PCA scores (Figures 2C, D). Patients with high PCA scores had a higher proportion of mortality, and patients who died had higher PCA scores than patients who were alive (Figures 2E, F). These results suggest that the better survival of the C2 subtype may be related to lower focal adhesion gene expression.

### 3.3 4-Genes signature is a robust prognostic factor for COAD

**FIGURE 3:**
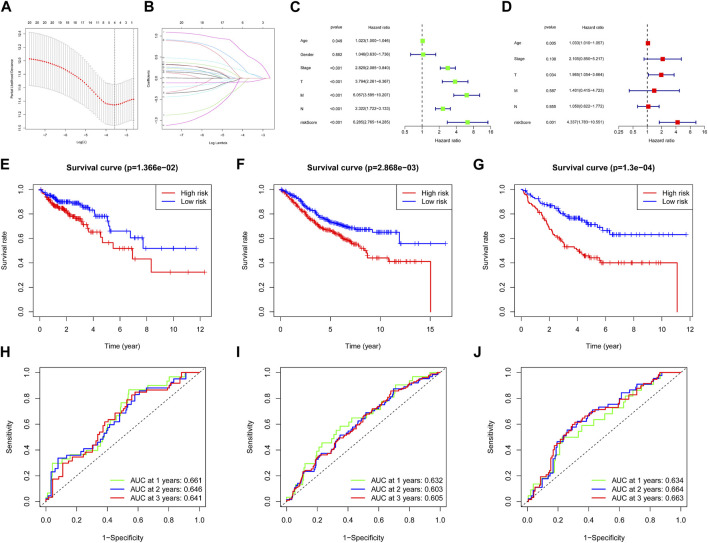
Construction of the adhesion molecules related risk signature model. **(A)** Partial likelihood deviance of variables revealed by the Lasso regression model. The red dots represented the partial likelihood of deviance values, the gray lines represented the standard error (SE), the two vertical dotted lines on the left and right represented optimal values by minimum criteria and 1-SE criteria, respectively. **(B)** Significance and hazard ratio (95% CI) values of OS-related ARGs in univariate Cox regression. **(C)** Independent prognostic-related factors screened out by COX regression analysis. **(D)** Survival analysis of two risk groups of the 4-gene signature in training cohort **(E)**, GSE17538 cohort **(F)** and GSE39582 cohort **(G)**. ROC analysis of two risk groups of the 4-gene signature in TCGA cohort **(H)**, GSE17538 cohort **(I)** and GSE39582 cohort **(J)**.

We used the COAD patients of TCGA as the training set. The 22 focal adhesion genes were further screened and collinearity was removed to obtain four focal adhesion genes, CAV2, FLT1, THBS3 and VAV3 through the LASSO-Cox algorithm (Figures 3A, B). Finally, a risk scoring model of *ß* (Coef) value multiplied by ARG score was established. We divided 343 patients into high-risk and low-risk groups using the median risk score as the cutoff. Univariate Cox regression showed that risk score and age, Stage, T, N, and M were closely related to OS (Figure 3C). We further performed multivariate Cox analysis and found that risk score was an independent prognostic factor in COAD patients (*p* < 0.001) (Figure 3D). The Kaplan–Meier curve showed that the OS of the low-risk group was significantly better than that of the high-risk group (Figure 3E). We obtained similar results using the same method on the GSE17538 (Figure 3F), GSE39582 (Figure 3G) test datasets. The model-predicted AUCs for 1-, 2-, and 3-year OS in the training group were 0.661, 0.646, and 0.641, validating the signature’s predictive performance (Figure 3H), we got similar results in the GSE17538 (Figure 3I), GSE39582 (Figure 3J) test datasets.

### 3.4 Patients with COAD in the high-risk and low-risk groups have different patterns of immune infiltration

**FIGURE 4:**
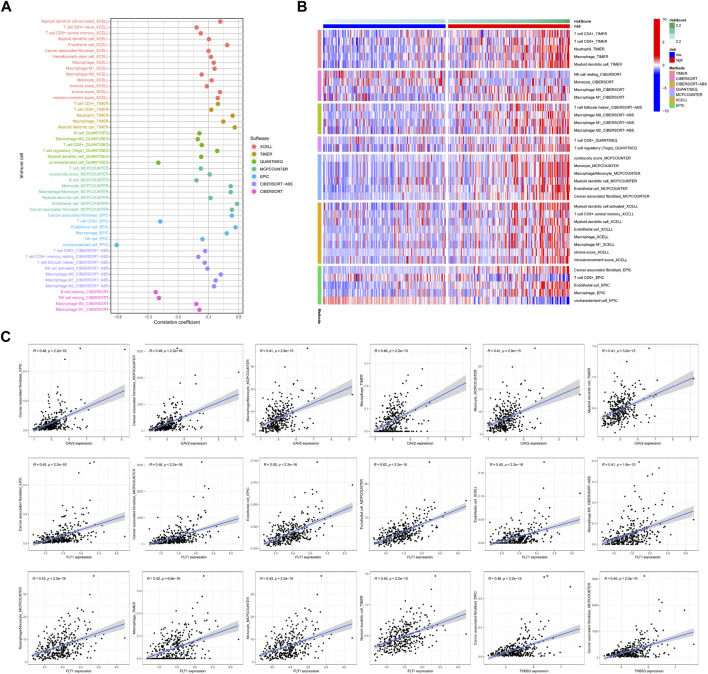
Tumor microenvironment analyses. **(A)** The relationship between the riskscore and 7 software scores in tumor tissues, *p* > 0.05 will be included in the bubble chart presentation. **(B)** Comparations between high-risk group and low-risk group in terms of 7 software scores. p > 0.05 will be included in the heat map presentation. **(C)** Correlation analysis of mRNA expression of genes in the model with immune scoring, only two or more software scores were included.

Our signature quantifies the risk of COAD patients well. To clarify why there is significant heterogeneity between the high-risk group and the low-risk group, we also used multi-software to compare the differences in immune infiltration between the high- and low-risk groups. We found that, the risk score is positively correlated with the infiltration of various immune cells, including T cells, DCs, and macrophages (Figure 4A). The heat map shows that high-risk patients have more infiltration of CD4 T cells, CD8 memory T cells and other immune cells (Figure 4B). While high-risk patients had worse prognosis, implying that immune escape may happened in patients of the high-risk group. We found that the genes CVA2, FLT1, and THBS3 in the model were all positively correlated with CAFs (Figure 4C), suggesting that adhesion molecules may remodel the immune microenvironment and mediate immune escape through CAFs in COAD.

### 3.5 Patients with COAD in the high-risk and low-risk groups are biologically unique

**FIGURE 5:**
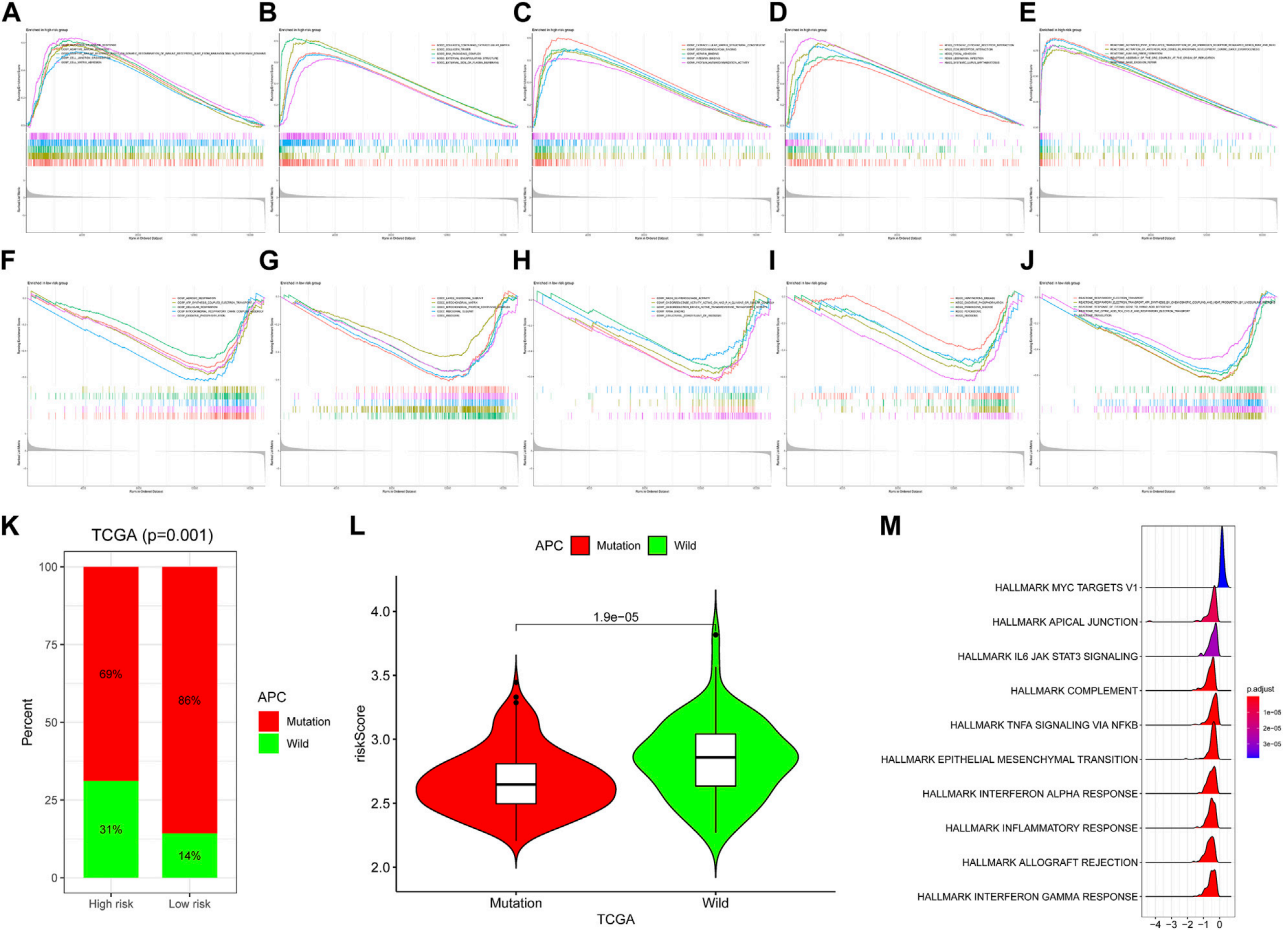
Functional enrichment analyses. In the high-risk group, **(A–C)** GO (BP, CC, MF), **(D)** Kyoto Encyclopedia of Genes and Genomes and **(E)** Reactome pathways that was enriched in **(F–H)** GO (BP, CC, MF), **(I)** Kyoto Encyclopedia of Genes and Genomes and **(J)** Reactome pathways that was enriched in the high-risk group of the TCGA-COAD dataset. Enriched in the high-risk group of the TCGA-COAD dataset. **(K)** Differences in the proportions of wild-type and mutant APC in the high- and low-risk groups. **(L)** Differences in wild-type and mutant risk scores. **(M)** Enrichment analysis of APC mutant over wild-type pathway based on hallmark gene set. Greater than 0 means that the pathway is significantly enriched in mutant and less than 0 means that the pathway is significantly enriched in wild-type.

To further investigate the significant heterogeneity between the high-risk group and the low-risk group, we conducted studies on related biological mechanisms and signaling pathways. We performed a gene set enrichment study (GSEA) based on GO (BP, MF, CC), Reactome, and Hallmark gene sets for high-risk and low-risk groups. In the high-risk group, signaling pathways or biological processes such as cell junction organization, DNA packaging complex, integrin binding and focal adhesion were enriched, while in the low-risk group, cellular respiration, ribosome, NADH dehydrogenase activity, oxidative phosphorylation and other signaling pathways Enrichment (Figures 5A–J), in order to illustrate the effect of mutations on the development of COAD, the APC mutation rate of our high-risk patients was lower than that of low-risk patients, and APC mutations were mostly enriched in low-risk patients (Figures 5K–L). Additionally, the hallmark myc targets v1 is upregulated in the mutant phenotype and all others are upregulated in the wild type (Figure 5M). Possible alterations in COAD biology mediated by APC mutations.

### 3.6 Patients in the high-risk group are better suited to receive immunotherapy

**FIGURE 6:**
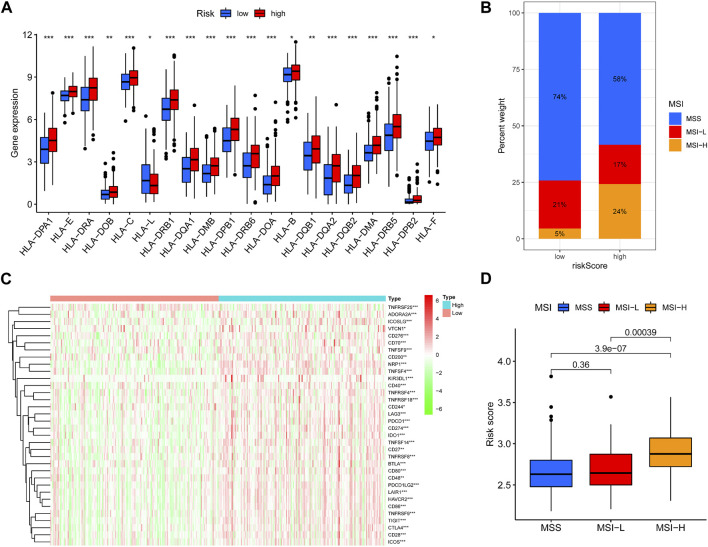
Predicting immunotherapy response. **(A)** Boxplots were used to display the expressions difference of HLA. **(B)** Differences in MSS-H, MSS-L, MSS ratios between high and low risk groups. **(C)**Heatmap were used to display the expressions difference of checkpoint. **(D)** Differences in MSS-H, MSS-L, MSS risk scores.

Since immunotherapy is an emerging treatment, and COAD has a low response rate to immunotherapy, we hope to guide the personalized immunotherapy of COAD through our risk model. We used MSI, HLA diversity, and immune checkpoint expression to jointly determine the choice of immunotherapy for patients in high and low risk groups. We found higher HLA expression in high-risk patients (Figure 6A). High-risk patients exhibited higher proportions of microsatellite instability-high (MSI-H) and lower proportions of microsatellite stable (MSS) (Figure 6B). The heatmap showed higher immune checkpoint expression in high-risk patients (Figure 6C). MSI-H was more enriched in high-risk patients (Figure 6D). These results suggest that high-risk patients may have high response rates to immunotherapy.

### 3.7 Expression landscape of genes at the transcriptional and protein levels in the model

**FIGURE 7:**
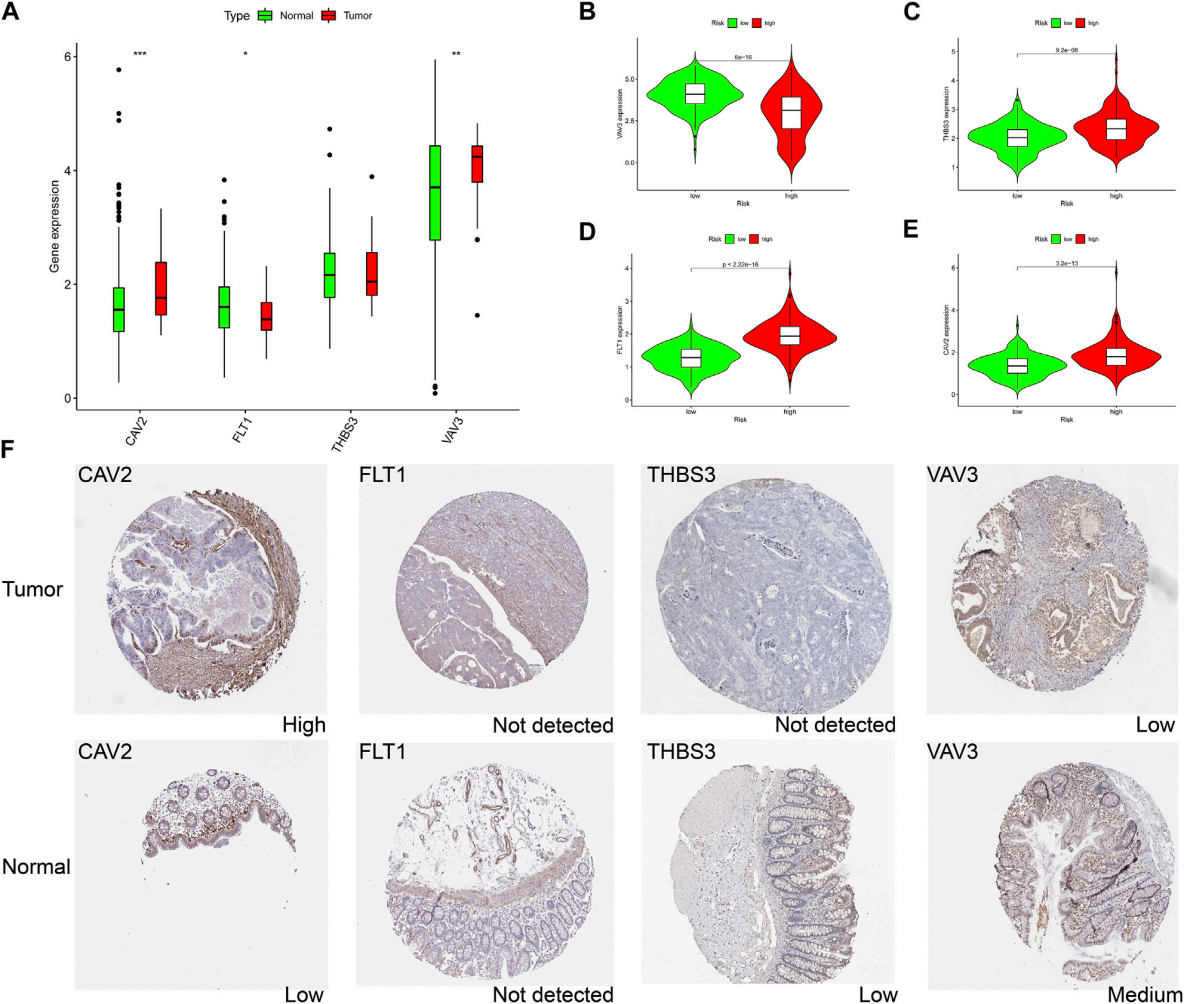
The expression landscape of genes in signature. **(A)** Differences in gene mRNA expression in signature in normal and tumor groups based on TCGA database. **(B–E)** Differences in gene mRNA expression in signature in high and low risk groups based on TCGA database. **(F)** Immunohistochemistry staining of the prognostic genes in COAD and normal liver tissues (the HPA database). ∗*p* < 0.05; ∗∗*p* < 0.01; ∗∗∗*p* < 0.001.

Combined with the immunohistochemical data from the database, we investigated the protein level changes of CAV2, FLT1, THBS3 and VAV3 in tumor samples and normal control samples. We found that CAV2 and VAV3 were significantly highly expressed in tumor samples (Figure 7A), whereas the expression levels of THBS3 and FLT1 were not significantly altered in tumor samples. In the high-risk group, VAV3 was lowly expressed and the remaining three genes were highly expressed (Figures 7B–E). Data in the HPA database suggested that CAV2 was highly expressed in the tumor, consistent with the transcriptional level, while the other genes were not expressed consistent with the transcriptional level (Figure 7F). These results suggest that changes in tumor transcriptome levels may not be consistent with changes in protein levels, and perhaps changes in protein levels also have an impact on prognosis of patients, which needs further verification.

## 4 Discussion

COAD is characterized by high recurrence rate and poor prognosis. Accurately predicting the prognosis of COAD patients is of great significance to guide their treatment. Focal adhesions act on the adhesion between cells and participate in the migration and progression of tumor cells. Past studies have shown that the focal adhesion gene FAK regulates tumor progression (Barker et al., 2013; Chen et al., 2014). Our study systematically investigated 199 focal adhesion genes to explore the function of focal adhesion in ACOD. Univariate analysis identified 22 focal adhesion genes associated with the prognosis of ACOD, among which, FAK was not included, suggesting that the role of FAK in ACOD is complex and may co-regulate tumor progression with other factors. The 22 focal adhesion genes we screened provide us with new important clues to study the function of focal adhesion in COAD. Furthermore, our findings suggest that the SRC, VAV3 genes are a protective factor for the prognosis in COAD, while the other 20 genes are risk factors.

In order to further study the function of focal adhesion in COAD, we compared the expression levels of prognostic focal adhesion genes in each sample, and innovatively established ARGP score, and finally identified 4 ARGs signature, CAV2, FLT1, THBS3, and VAV3, we found that patients in the low-risk group had the better OS than that in the higher-risk group. Past studies have suggested these four genes involved in tumor development. CVA2 is an encoded protein, which is the main component of the inner surface of vesicles and the invagination of plasma membrane, and is involved in basic functions such as lipid metabolism and signal transduction (Liu et al., 2020). In a large-scale exome-wide correlation study of pancreatic cancer patients, it was found that genetic variation and high expression of CVA2 promote pancreatic cancer progression and are associated with poor prognosis (Zhu et al., 2021). FLT1 is a gene that regulates angiogenesis, its expression is upregulated in colorectal cancer, and it is associated with poor prognosis of patients (Mohammad Rezaei et al., 2019), which is consistent with our results. THBS3 is a gene of the Thrombospondins (Thbs) family in vertebrates. It is one of the family of cellular glycoproteins and plays an important function in the interaction between cells and matrix (Schips et al., 2019). THBS3 is also thought to be involved in immune processes such as antigen presentation and T cell differentiation (Deng et al., 2021), and may also play a role in tumor immunity. VAV3, as a guanine nucleotide exchange factor, regulates the activity of Rho/Rac family GTPases. Activation of VAV3 can promote tumor metastasis, in non-small cell lung cancer (Chen et al., 2020). However, VAV3.1, a specific truncation variant of VAV3, is highly expressed in endometrial carcinoma but is not associated with poor patient prognosis and tumor stage (Boesch et al., 2018), and our study found VAV3 to be a protective factor for COAD.

External dataset validation and internal hierarchical validation prove that the model has robust predictive performance. It enables clinicians to more accurately and effectively assess patient survival. The use of focal adhesion gene pairs to construct a prognostic model may be an important complement to predict the prognosis of COAD. Furthermore, we explored the potential functions of 4-gene ARGs in COAD by GSEA. In the high-risk group, signaling pathways or biological processes such as cell junction organization, DNA packaging complex, integrin binding, and focal adhesion were enriched, and these signals were associated with tumor cell proliferation and migration (Dawson et al., 2021), while in the low-risk group, cellular Respiration, ribosome, NADH dehydrogenase activity, oxidative phosphorylation and other signaling pathways are enriched, and these mitochondrial and respiration-related pathways are related to the function of immune cells (Boreel et al., 2021), suggesting that focal adhesion genes may affect the prognosis of COAD by regulating the immune microenvironment. A previous study has shown that focal adhesion genes can reduce tumor cell invasion and attenuate host cell activation in the tumor microenvironment by activating cancer-associated fibroblasts in the tumor microenvironment (Barker et al., 2013). The combination of four genes may also regulate the tumor immune microenvironment of COAD patients by regulating cancer-associated fibroblasts.

However, our study also has some limitations. This is a retrospective study with data from the TCGA and ICGC databases, lacking information on treatment and recurrence records. Our conclusions need to be validated *in vivo* or *in vitro* experiments and prospective clinical studies.

## 5 Conclusion

In conclusion, we systematically investigated the effect of focal adhesion genes on the prognosis of ACOD for the first time, and found a 4-ARGs with prognostic value in COAD patients. Our study proposes predictive models and biomarkers for COAD patients and also provides a basis for further mechanistic and therapeutic studies.

## Data Availability

The original contributions presented in the study are included in the article/Supplementary Material, further inquiries can be directed to the corresponding author.
